# Electronic cigarette use in China: Awareness, prevalence and regulation

**DOI:** 10.18332/tid/105393

**Published:** 2019-04-16

**Authors:** Wenyuanyue Wang, ZiAn He, Nannan Feng, Yuyang Cai

**Affiliations:** 1 School of Public Health, Shanghai Jiao Tong University School of Medicine, Shanghai, China; 2 NHC Key Laboratory of Health Economics and Policy Research, Shandong University, Jinan, China

**Keywords:** electronic cigarettes, awareness, prevalence, regulation, China

## Abstract

**INTRODUCTION:**

E-cigarettes were invented in China, and most of the world’s e-cigarettes have been produced in China. However, awareness and use of e-cigarettes in China are lower than in Europe and America. Against the backdrop of the increasing use of e-cigarettes, the supervision of e-cigarettes in China has been almost non-existent.

**METHODS:**

A literature search was carried out in five popular Chinese and English databases. These databases were PubMed, EMBASE, the Cochrane Library, China national knowledge infrastructure (CNKI), and Wan-fang database.

**RESULTS:**

Twenty-one studies were identified, and a comprehensive analysis of e-cigarette awareness ratio was conducted for different regions of China and according to gender, age group, and smoking status. We also examined e-cigarette use and associated risk factors, and the relationship between e-cigarette use and smoking cessation. In 2015, the prevalence of ‘ever use’ and ‘current use’ of e-cigarettes in China were 3.1% and 0.5%, respectively. The review indicates that the awareness ratio of e-cigarettes was about 66% in Hong Kong, whereas the ratio for Tianjin was lower (43.6%). Online sales were the main channel for selling e-cigarettes to 80% of the users. Awareness of e-cigarettes has been increasing in China. Awareness was higher in men compared to women in all age groups. Nevertheless, e-cigarette use in China was lower than in developed countries. E-cigarette users were more likely to try to quit smoking, but the relationship between e-cigarette use and smoking cessation is still unclear. Governance is necessary for e-cigarette use and marketing effort.

**CONCLUSIONS:**

This study investigated the awareness and use of e-cigarettes in China and the existing regulations for e-cigarette use and marketing. The lack of regulations for e-cigarette use and the unrestricted practice encourage the increase in adoption of e-cigarettes and misconceptions of the benefits of using e-cigarettes. Hence, it is crucial that the government of China prioritize the establishment and implementation of regulations for e-cigarette use and marketing.

## INTRODUCTION

Although electronic cigarettes (e-cigarettes) were invented in China, the majority of e-cigarettes users reside in Europe and the United States^1^. The prevalence of ‘ever use’, ‘currently use’ (at least 1 of the last 30 days), and ‘regular use’ (at least 20 of the last 30 days) of e-cigarettes in the US were 7.7%, 2.1%, and 0.9%, respectively^2^. In Europe, the prevalence of e-cigarette ‘current use’ was 1.8%^3^. The 11.6% prevalence of ‘ever use’ of e-cigarettes in Europe is higher than in the US^3^. However, in China where the e-cigarette originated, the prevalence of e-cigarette use is low^4^. According to the China Adult Tobacco Survey in 2015, 40.5% of adults aged 15 years and older had heard of e-cigarettes and 3.1% had ever tried them^5^. Hence, in this work we have collected, analyzed, and synthesized the existing literature on the Chinese population’s awareness and use of e-cigarettes. In our study, the ‘awareness of e-cigarettes’ is defined by the first incidence the individuals heard of e-cigarettes. The awareness ratio characterizes the proportion of the population that has heard of e-cigarettes.

E-cigarettes, most accurately known as ‘electronic nicotine delivery systems (ENDS)’, consist of a lithium battery, smoke bomb (containing volatile liquid), pressure sensor, control circuit board, and light emitting diode. When the volatile liquid of the smoke bomb is heated, the device produces smoke fog. Depending on the preference of the users, nicotine and other chemicals may be added into the volatile liquid. The operational principle of e-cigarettes mimics that of traditional cigarettes^6^. Hon Lik, a Chinese pharmacist, is widely considered to be the inventor of the first generation of e-cigarettes with the first nicotine-based e-cigarette produced in 2003. The first generation of e-cigarettes was introduced to the Chinese market in 2004 and exported since 2005^7^. In 2015, China produced about 80% of the world’s e-cigarettes^8^, with a 33% increase in sales in 2015 alone^9^.

Compared with people in the European Union, the US and other countries, Chinese know less about the use of e-cigarettes than people of other Asian countries^10^. However, the International Trade Center (ITC) China survey revealed that the percentage of smokers who had heard of e-cigarette rose from 29% (wave 3; 2009) to 60% (wave 5; 2014). Similarly, the percentage of smokers who had tried e-cigarette surged from 2% (wave 3; 2009) to 11% (wave 5; 2014)^11^. These surveys suggest that both the number of individuals who are aware of e-cigarettes and the number of individuals using e-cigarettes are rising.

Despite the rapidly increasing use of e-cigarettes in China, the governance of e-cigarette use is still at an early stage compared to other countries that have enacted relatively clearer regulations related e-cigarette use and supply^12^. In China, the only regulation of e-cigarette use was published by the State Administration of Market Supervision and Administration and the State Tobacco Monopoly Bureau in 2018. This regulation was stated in the ‘Circular of the State Administration of Market Supervision and Administration and the State Tobacco Monopoly Bureau on the Prohibition of the Sale of Electronic Cigarettes to Minors’. Hang Zhou city formulated local regulations of e-cigarettes to manage the use of e-cigarettes in smoke-free places^13^. Until today, national law or regulation, published by NPC (National People’s Congress) and Standing Committee of NPC and the State Council, to control e-cigarette use is still lacking. Nevertheless, the government of China realizes the importance and urgency of controlling e-cigarette use nationally^14^.

## METHODS

### Search strategy

A literature search was carried out in five popular electronic Chinese and English databases on 1 December 2018. These databases were PubMed, EMBASE, the Cochrane Library, China national knowledge infrastructure (CNKI), and Wan-fang database. The queries combed through the publications in the databases from their inceptions to 30 November 2018. We used subject terms to search in the databases and manually retrieved the list of references.

In the Chinese databases, we used the subject heading search, and the search term was expressed as ‘电子烟’ (i.e. e-cigarette). In addition, the search field included the title, abstract, keyword, and subject word. In the English databases, we used Medical subject Headings (MeSH), and the search term was ‘electronic nicotine delivery systems’ and ‘e-cigarette’.

### Inclusion and exclusion criteria

Two investigators screened the literature independently based on the inclusion and exclusion criteria (Table 1). The purpose of literature screening was to filter out studies that did not match the inclusion criteria.

**Table 1:** Inclusion and exclusion criteria used in literature screening

Inclusion criteria	Exclusion criteria
Language is Chinese or English	Language is not Chinese or English
Document type is a published journal article	Document type is not a published journal article
The subject of literature research is ‘e-cigarette’	The subject of literature search does not include ‘e-cigarette’ and only related to smoking and tobacco
Focus on research in China, or research that reflects the status and progress of e-cigarettes in China	The literature on the status and progress of e-cigarettes in China is not mentioned in the study
The research content includes any of the following aspects:	The research content does not contain any of the following items and is only related to the production processes and chemical processes:
Epidemiological investigation	Epidemiological investigation
Crowd survey	Crowd survey
Quit smoking	Quit smoking
Regulatory policy	Regulatory policy
Product sales and promotion	Product sales and promotion

### Study selection

We searched five Chinese and English databases and obtained 4075 articles initially. We then imported all 4075 citations into EndNote (Clarivate Analytics, X8) and deleted duplicated documents.

After eliminating the duplicates, we read the headings and abstracts and conducted a preliminary screening of the literature based on the inclusion and exclusion criteria (Table 1). We excluded papers that did not meet the inclusion criteria. For the papers that were retained after the first round of screening or papers that could not be decided on at this stage, we read the full text and performed the second round of screening according to the inclusion and exclusion criteria (Table 1).

**Figure 1:**
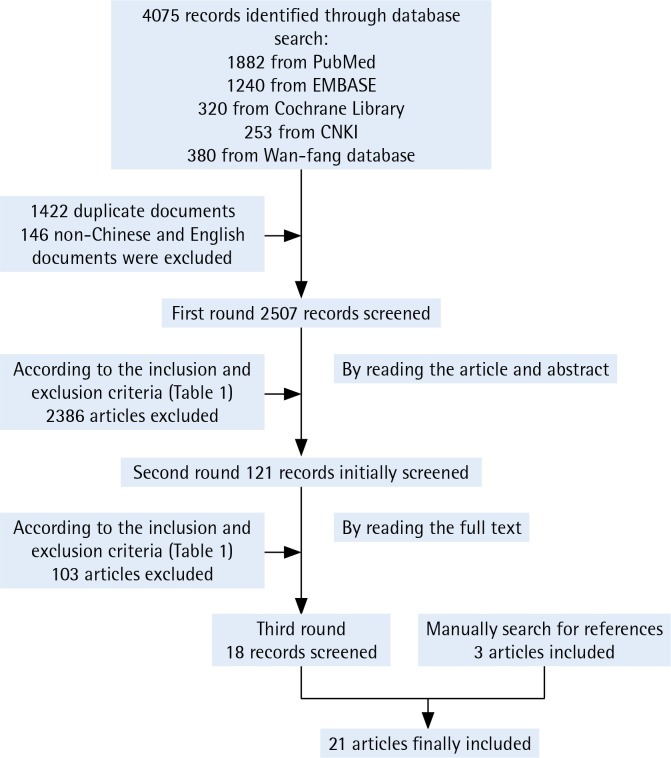
Filtering procedure for article inclusion

When disagreement appeared during the screening process, we discussed and exchanged views with another independent investigator in order to reach an agreement. Finally, we further filtered the papers that failed to meet the inclusion criteria and kept 21 papers (Figure 1).

### Quality assessment

As some of the included literature adopted a combination of several research methods in a single study and some studies had narrowly defined research objectives for the designated disciplines, we could not find an evaluation framework that was suitable to evaluate such a diverse body of literature. Therefore, we adopted the evaluation approach proposed by Moola et al.^15^ because this approach evaluates six general aspects of the 21 papers. These aspects are: 1) clarifying the research purpose clearly; 2) clarifying the research method clearly; 3) explaining the study subjects and their settings clearly; 4) clarifying the source of data clearly; 5) whether the results are measured in a scientifically valid manner; and 6) the logic and conviction between conclusions and analysis.

Papers that matched at least five aspects suggested by Moola et al. were regarded as high-quality literature. Papers that fulfilled three or fewer aspects were considered low-quality documents. The remaining papers were considered as medium-quality literature.

### Data extraction and analysis

According to the inclusion and exclusion criteria (Table 1), we extracted information from 21 retained papers. For the 13 population and epidemiological surveys of these 21 papers, we extracted relevant information by studying the research methods and the characteristics of the sample population in detail.

We extracted the data independently and reached consensus through discussions. The extracted data included author/year, investigation method, study area, sample, statistical method, report result and quality (Appendix 1). For the remaining 8 papers, the extracted data included author/year, study area, literature results, and quality (Appendix 2).

## RESULTS

### Awareness of e-cigarettes in China

We measured the awareness of e-cigarettes among the sample populations in the included literature with a closed-ended question: ‘Have you heard of e-cigarettes?’. Individuals in the sample population in the included literature who answered ‘yes’ were considered as being aware of e-cigarettes. The total number of individuals who answered ‘yes’ divided by the sample population represents the ‘awareness ratio’.

#### High awareness of e-cigarettes

According to the 2015 China Adult Tobacco Survey, 40.5% of adults aged 15 years and older had heard of e-cigarette. Moreover, the International Tobaco Control (ITC) China survey revealed that the percentage of smokers who had ever heard of e-cigarettes rose from 29% (wave 3; 2009) to 60% (wave 5; 2014)^11^. Recently, a study in Hong Kong indicated that the awareness ratio of e-cigarettes is about 66% and seems to be rising^16^, while in Tianjin, the e-cigarette awareness ratio was slightly lower (43.6%) and the awareness ratio of e-cigarettes among the 25–44 years age group was the highest among all the age groups^17^. In China, the awareness ratio of e-cigarettes among adolescents (aged 12–18 years) was 89.52% with the e-cigarette awareness ratio of current cigarette smokers (95.93%) and former cigarette smokers (93.23%) both higher than for non-smokers (85.75%)^18^. The study further suggests that most adolescents knew about e-cigarettes through the internet and television^18^. About 69.8% of cigarette smokers in Beijing (age ≥20 years) had heard of e-cigarettes from their friends and tobacco retailers besides the advertisements on television and the internet^19^. In Hong Kong, 82.6% (95% CI: 80.2–84.9%) of cigarette smokers had heard of e-cigarettes^20^.

Several studies focused on the awareness of e-cigarettes among junior high school students because they are susceptible to online advertisements. One survey in Guangxi province showed that the e-cigarette awareness ratio among junior high school students was 46.71%, consistent with the results of the national survey on e-cigarette awareness (45%)^21,22^. The awareness ratio of e-cigarettes among adolescents in Hong Kong (71.1%) was higher than in mainland China^23^, and close to the level of awareness among the adults in Hong Kong (75%, 2014)^16^.

#### Higher awareness of e-cigarettes in the male population

Several studies found that the awareness of e-cigarettes was higher in men compared to women in all ages, as expected considering that men smoke more often than women and they can learn about e-cigarettes through more sources^11,16,17,21,22^. The survey also indicates that the awareness of e-cigarettes among youth was influenced by social and individual factors. Individuals that have close friends who smoked were more likely to know about e-cigarettes than those who did not have close friends who smoked (OR=1.66). Individuals who had smoking experience (OR=1.72) or individuals who paid attention to tobacco advertisements were also more likely to be aware of e-cigarettes (OR=1.55)^22^.

#### Higher e-cigarette awareness when more educated

A survey carried out in Tianjin showed that the awareness of e-cigarettes was positively related to the level of education. The awareness ratio of e-cigarettes was higher among the population with more education^17^. This trend was also similar among cigarette smokers^20^. Furthermore, younger people and people with higher education were more likely to be aware of e-cigarettes^11^.

### E-cigarette use and related risk factors

In the included literature, the ‘use’ of e-cigarettes was broken down by ‘ever use’, ‘current use’, and ‘use in past 30 days’. The first category was answered by the question: ‘Have you ever tried e-cigarettes?’. The number of people who answered ‘yes’ divided by the sample population represents the prevalence of ‘ever use’. The second category ‘current use’ was measured by the question: ‘Do you use e-cigarettes now?’. The third category was measured by the question: ‘Did you use e-cigarettes in the past 30 days?’. We distinguished these three categories by seeking the questions and answers in the included literature that contained the phrase ‘current’ and ‘in the past 30 days’. The prevalence of ‘current use’ and ‘use in past 30 days’ are defined in a similar way.

#### Low e-cigarette use prevalence

E-cigarette use in China was lower than that in developed countries such as the US and European countries^2,3^. According to the 2015 China Adult Tobacco Survey, 3.1% of adults aged 15 years and older had ever tried e-cigarettes and the current use prevalence of e-cigarettes was 0.5% (Table 2). Although the report suggests that most people used e-cigarettes occasionally^5^, the percentage of cigarette smokers who had ever tried e-cigarettes still rose from 2% (wave 3; 2009) to 11% (wave 5; 2014)^11^.

**Table 2:** Prevalence (%) of e-cigarette use amongst adults, adolescents and smokers

		Ever use	Current use	Use in past 30 days
Adults	2015 China Adult Tobacco Survey	3.1	0.5	
	Tianjin	2.3	0.5	
	Hong Kong	2.3		
Adolescents	China	26.44		
	GuangXi	8.5		1.2
	Hong Kong	8.9		1.1
	Taiwan			27.46
Smokers	Beijing	12.8		
	Hong Kong	13.3		

The survey conducted in Tianjin showed that 2.3% of the respondents had ever used e-cigarettes and only 0.5% of them were still using e-cigarettes. These findings are consistent with the results of the national survey^17^. Similarly, in Hong Kong, e-cigarette use among adults was 2.3% (‘ever use’) with men using e-cigarettes more often than women (p=0.03). People between the age of 15 and 29 years were more likely to use e-cigarettes (p=0.002). The current cigarette smokers had the highest prevalence^16^. The use of e-cigarettes was different among different ages. A study showed that 26.44% of Chinese adolescents had used e-cigarettes (including one-time use)^18^. Another study conducted in Hong Kong among adolescents found that e-cigarette use was about 8.9% (‘ever tried’)^23^, which was higher than for the adult population (between age 15 and 65 years). The e-cigarette ‘use in the past 30 days’ among adolescents in Hong Kong was about 1.1%^24^. Among the younger students in Guang Xi junior high school, the use of e-cigarettes was relatively low, at 8.5% for ‘ever tried’, and 1.2% for ‘use in the past 30 days’^22^. Surprisingly, the e-cigarette use ‘in the past 30 days’ among adolescents in Taiwan increased rapidly to 27.46%^25^. In general, cigarette smokers had a higher use prevalence of e-cigarettes. In Hong Kong, 13.3% (95% CI: 11.3–15.5%) cigarette smokers had ever used e-cigarettes^20^. In Beijing, 122 (12.8%) cigarette smokers had ever used e-cigarettes^19^. These findings imply that adolescents used e-cigarettes more often than adults.

#### Influencing factors of e-cigarette use and frequency

Research shows that gender and smoking status can influence e-cigarette use^17^. Meanwhile, the study in Hong Kong suggests that men used e-cigarettes more often than women (p=0.03)^16^. In the survey of e-cigarette use among junior high school students in Guangxi, the multivariable logistic regression analysis shows that the prevalence of e-cigarette use was 2.422 times in men than in women (p<0.01). Besides, age, academic performance (such as grades) and peer smoking could also influence e-cigarettes use (p<0.01)^21^.

In Beijing, 122 (12.8%) cigarette smokers, 114 men and 8 women, had ever used e-cigarettes^19^. Among the cigarette smokers, the prevalence of e-cigarette use among urban smokers (18.2%) was significantly higher than among rural smokers (9.3%) (p<0.001). However, the intention of e-cigarette use among rural smokers (25.7%) was higher than that of urban smokers (17.2%) (p=0.022)^19^. The study also investigated the attitudes of e-cigarette use among cigarette smokers, and the results show that 57.0% of cigarette smokers had heard of e-cigarettes but not used e-cigarettes, 22.6% would like to try, and 12.1% did not give a clear attitude. Amongst the 65.3% cigarette smokers, the percentage who did not want to use e-cigarette because of they did not intend to quit smoking was 40.7%, distrusted of the effects of e-cigarettes was 27.8%, worried about the safety of e-cigarettes was 19.7%, concerned about prices was 3.7% and others (8.1%)^19^. A similar result was found in the 2015 China Adult Tobacco Survey, in which younger people, male, and urban residents, preferred using e-cigarettes^5^.

One study about urban male tobacco cigarette smokers in China revealed that the younger, with higher education, those who attempted to quit smoking in the past year, and who smoked more cigarettes per day, were more likely to use e-cigarettes^26^.

#### Motivations to use e-cigarettes

Among Chinese adolescents, most used e-cigarettes because they wanted to avoid the harms from directly inhaling the chemicals in traditional cigarettes (44.63%) and indirectly inhaling the chemicals in secondhand smoke (26.30%) produced by other traditional cigarette smokers, while 24.26% wanted to quit smoking by using e-cigarettes. Some respondents used e-cigarettes because they thought it was fashionable (15.37%) or simply because they were curious about e-cigarettes (25.56%). A small number of e-cigarette users thought that e-cigarettes were cheaper than traditional cigarettes and could be used in smoke-free places^18^.

For slightly different purposes, adults in Hong Kong used e-cigarettes for curiosity (47.4%), fashion (25.8%) and quitting smoking (13.6%)^16^.

### The marketing of e-cigarettes

We searched for the keyword ‘e-cigarettes’ on the Taobao website (China’s largest e-commerce platform) and found that more than 10000 e-cigarette products were sold monthly. On the ‘Sales’ webpage, the following information was presented: 1) e-cigarettes were healthier than traditional cigarettes; 2) e-cigarettes could help smokers quit traditional cigarettes; 3) e-cigarettes had many flavors; and 4) e-cigarette smoking was permitted in non-smoking areas. On the ‘Ingredients’ webpages, marketers disclosed that e-cigarettes contained propylene glycol, nicotine salt, and essence.

Yao et al.^27^ found that most websites (16 out of 18) claimed that e-cigarettes were healthier and excluded hazardous ingredients such as nicotine. Also, 14 websites indicated that e-cigarette use might prevent secondhand smoking. Secondhand smoking refers to the inhaling of smoke emitted from the burning of tobacco products involuntarily. Twelve websites claimed that e-cigarettes could help cigarette smokers quit smoking and that they were allowed to be used in non-smoking areas. Eight websites indicated that e-cigarettes were cleaner than traditional cigarettes, while 7 websites hinted that e-cigarettes could enhance users’ social status^27^.

Similar to Western countries, 22 markets of e-cigarettes in China targeted the young generation. The Chinese marketers promoted their products as fashion accessories with the e-cigarette’s modern and stylish design and multiple flavors. The e-cigarettes were seen as part of the fashion of pop icons among the youth. The marketing companies launched competitions with appealing prizes and hired young, attractive women to boost e-cigarettes sales^28^.

In China, e-cigarette sales reached 4.09 billion RMB (about 589 million USD) in 2017, an increase of 25.3% year-on-year in the consumer market. Online sales of e-cigarettes accounted for around 80% of the consumer market and store sales 14%, while sales from other marketing channels represented a small market share^29^.

### E-cigarette use and smoking cessation

Recently, a study in Beijing showed that e-cigarettes could help cigarette smokers quit smoking or reduce smoking^19^. The study found that 71.0% of cigarette smokers expected to quit smoking by using e-cigarettes. In fact, 8.3% of cigarette smokers had quit smoke successfully, 47.1% cigarette smokers used fewer cigarettes, and 15.1% cigarette smokers used the first e-cigarette later in the morning compared to the time when they smoked the first traditional cigarette in the morning. In this study, most cigarette smokers (84.0%) believed that e-cigarettes were not addictive or less addictive than traditional cigarettes. More than 70.0% of cigarette smokers believed that e-cigarettes were healthier and less risky than traditional cigarettes^19^. Chen et al.^25^ suggest that 71% of e-cigarette users had tried to quit smoking in the past year compared to 69% of non-e-cigarette users, with a statistically significant difference (p<0.05)^25^. Of the people who used e-cigarettes, 39.47% had attempted to quit smoking in the past 12 months, significantly higher than those who had never used e-cigarettes (13.98%) (p<0.01)^17^. In the Wang et al.^18^ study, 36.02% of cigarette smokers had tried to quit smoking by using e-cigarettes. However, only 13.54% of cigarette smokers quit smoking successfully. They used multivariable logistic regression to analyze the relationship between e-cigarette use and smoking cessation behavior. The study indicated that using e-cigarettes was related to smoking cessation attempts (OR=1.6, p<0.05). E-cigarette users were more likely to try to quit smoking, but the relationship between e-cigarette use and smoking cessation was still unclear^18^.

The relationships between the use of e-cigarettes and smoking cessation, attempt at smoking cessation, and smoking reduction, are inconclusive. Some studies report a positive relationship^17-19,25^, i.e. the use of e-cigarettes increases the proportion of people who quit smoking, attempt to quit smoking, and smoke less frequently. In contrast, some research indicates an inverse relationship between the use of e-cigarettes and smoking cessation, attempt at smoking cessation, and smoking reduction^20,22,24,30^.

In the study of Xiao et al.^22^, users of e-cigarettes were more likely to use tobacco products in the next 12 months (OR=7.0), more likely to use tobacco products provided by good friends (OR=5.1), and more likely to smoke (OR=14.6). One study among adolescents in Hong Kong showed that e-cigarette use was significantly correlated with increased smoking and smoking immediately after waking (p<0.001), but no remarkable correlation with smoking cessation behavior (p>0.05)^24^. A similar result was presented in a study among the Community-Recruited cigarette Smokers in Hong Kong^20^. The study showed that e-cigarette use is associated with lower levels of intention to quit and has no association with attempts to quit (p=0.45 for trend)^20^. Another study among cigarette smokers in Hong Kong showed that using e-cigarettes failed to predict self-reported point prevalence abstinence (AOR=0.99, 95% CI: 0.57– 1.73), biochemically validated quitting (AOR=1.22, 95% CI: 0.64–2.34), cessation attempt (AOR=0.74, 95% CI: 0.48–1.14), or smoking reduction (AOR=0.89, 95% CI: 0.54–1.47)^30^.

Significantly, Zhao et al.^26^ declared that the male respondents who attempted to smoke and smoked more cigarettes per day were more likely to use e-cigarettes. This finding may seem contradictory, but it may imply that the male respondents choose e-cigarettes to help them to quit smoking initially, but unexpectedly, e-cigarettes prompted them to smoke more.

### Supervision of e-cigarettes market

In China, e-cigarettes are not medicines, health products, medical devices, or tobacco^31^. The control of e-cigarette use by the Chinese government is almost non-existent. There is no law and administration regulation published by NPC (National People’s Congress) and Standing Committee of NPC and the State Council for e-cigarettes^29^. The only regulation published by State Administration of Market Supervision and Administration and the State Tobacco Monopoly Bureau on 28 August 2018, was in the ‘Circular of the State Administration of Market Supervision and Administration and the State Tobacco Monopoly Bureau on the Prohibition of the Sale of Electronic Cigarettes to Minors’^14^.

Considering the rapid increase of e-cigarette consumption, some areas in China have already established local e-cigarette use regulations. Hang Zhou city prohibits using e-cigarettes in non-smoking areas^13^.

When searching ‘electronic cigarettes’ in the ‘National public service platform for standards information’, we did not find any published guidelines for e-cigarette use. The search results only show two national guidelines that are pending for approval. One was ‘Electronic Cigarette’ and the other was ‘E-liquid—Determination of nicotine, propylene glycol and glycerol—Gas chromatographic method’^32^.

Additionally, some studies report that the nicotine concentration in the e-cigarette products was inconsistent with the label^33^. However, ‘Guang Ming Daily’, a national, comprehensive and influential daily newspaper directly under the Central Committee of the Communist Party of China with a worldwide readership, reports that some e-cigarettes had high nicotine content, carcinogens, and other toxic chemicals. The high concentration of harmful chemicals in e-cigarettes may cause more harm to humans than traditional cigarettes^34^. One study in Hong Kong showed that 71.1% of the residents supported the prohibition of e-cigarette promotion and advertisement, 81.5% supported the banning of e-cigarette use in smoke-free venues, 93.9% were in favor of banning the sale of e-cigarettes to minors, and 80.9% were in favor of sale restriction on nicotine-free e-cigarettes. Over half (57.8%) supported all four regulations^35^. From this study, we learned that most of the residents were in favor of some form of legislation targeting e-cigarette use and sales.

## DISCUSSION

A meta-analysis of 28 studies from 2009 to 2014 showed that the global average e-cigarette awareness ratio was about 61.2%^36^. According to the ‘Attitudes of Europeans towards tobacco and electronic cigarettes’ report issued by the European Commission in 2017, the awareness ratio of e-cigarettes in European Union (EU) countries increased from 69% (2012) to 84% (2017)^37^.

According to the China Adult Tobacco Survey in 2015, 40.5% of adults aged 15 years and older had heard of e-cigarettes^5^. The review of e-cigarette studies at the local level, such as Tianjin and Beijing, demonstrates that the Chinese e-cigarette awareness ratio is lower than the global and average European Union awareness ratios. The awareness ratio of e-cigarettes is slightly different amongst the various regions of China. Differences were predominantly caused by the diversity of economic development and culture in the different regions. For example, the awareness ratio of e-cigarettes among adolescents and adults in Hong Kong^16^ is about 70%, which is significantly higher than in mainland China (40.5%)^5^.

The awareness of e-cigarette ratio is also affected by the characteristics of the population. The distribution characteristics of awareness ratio can be expressed as: the awareness ratio of cigarette smokers is higher than that of non-smokers^18,22^; the ratio for males is higher than for females^16,17,22^; the ratio of online surveys^18^ is higher than of offline surveys^22^. These distribution characteristics are consistent with the conclusions of other international studies. In a study of e-cigarettes among adults in the US, awareness ratio was higher among adults aged 18–34 years, males, Whites, participants with some college education, individuals with household incomes above $75000, and those living in the Midwest^38^. In an Italian study, the awareness ratio was lowest among women, the elderly, those with low education, and non-smokers^39^. Our results illustrate that the above might correlate with the hypothesis that populations with higher awareness ratio are more accessible to the advertisement of tobacco.

From a meta-analysis of 67 surveys on the use of e-cigarettes, the overall estimate of e-cigarette use prevalence was 16.8%^36^. A survey of the use of e-cigarettes among US high school students showed that the prevalence of the past 30 days use of e-cigarettes increased ten-fold from 1.5% in 2011 to 16.0% in 2015, exceeding the proportion of traditional cigarettes^40^. By contrast, the use of e-cigarettes in China is still relatively low (3.1%)^5^, but its growth trend cannot be ignored^11^. A large rise in prevalence of e-cigarette use^11^ combined with a significant smoking population^4^ has the potential for a e-cigarette use to soar in China in coming years.

Several surveys reported that the main reasons for using e-cigarettes are that it is fun, enjoyable and cool^41-43^. These findings are consistent with survey results in Hong Kong^16^. However, most people, especially cigarette smokers in mainland China, perceived the use of e-cigarettes as less harmful than traditional cigarettes. Also, most people saw e-cigarettes as an alternative to traditional cigarettes that may help them to quit smoking^18,19^. As for adolescents and young adults, most were attracted by novel flavors and the fashionable design of e-cigarettes. Some cigarette smokers believed in advertisements that e-cigarettes did no harm their health and might help them quit smoking.

### Are e-cigarettes as harmless as advertisements say?

Kim et al.^44^ mention that the advertisements of e-cigarettes might remind people to smoke, and subsequently they fail to quit smoking. In our survey, one study suggested that the use of e-cigarettes might help adults quit smoking^19^. Some studies show that using e-cigarettes were correlated to willingness to quit smoking^17,18,25^. Other studies reveal that e-cigarette users are more likely to smoke^20,22,24,30^. In 2014, the World Health Organization (WHO) revealed that e-cigarettes had health risks to users and non-users. Besides, there was insufficient evidence showing that e-cigarettes could help quit smoking. One major concern is the conflicting interests in maximizing the potential benefits for smokers while increasing the possibility of encouraging the initiation of nicotine consumption among the non-smokers, especially the younger population. Such concerns are referred to as the gateway and renormalization effects^45^. The alarming fact that increasing trends in the awareness ratio and e-cigarette use prevalence among adolescents exceed those of adults demands immediate attention from the public and policymakers. From the studies of Wang et al.^24^ and Xiao et al.^22^, we find that the use of e-cigarettes may increase the likelihood of cigarette-smoking initiation among never cigarette smoking adolescents.

The myths of e-cigarettes create a distorted image of e-cigarettes. Therefore, the government should increase the public’s knowledge of e-cigarettes through health promotion activities and regulate e-cigarette advertisements to protect adolescents and young adults from the influence of misleading information and thus from forming wrong perceptions about e-cigarettes.

In China, e-cigarettes are not drugs, health products, medical devices, or tobacco products, in contrast to the United States where the FDA has classified e-cigarettes as tobacco products. The government of the United States limits the supply of e-cigarettes to consumers^46,47^; in Europe, the European Union Commission revised its regulations on tobacco products on February 2014 to prohibit e-cigarette manufacturers and traders from advertising their products^48^. However, in China, e-cigarette marketing and advertisement companies play an active role in fueling the popularity of e-cigarettes. The E-commerce platforms are the main channel for e-cigarette sales. So far, there is no regulatory measure of any impact on the circulation of e-cigarette products in the Chinese market^29^.

Our recommendation, for tackling the increasing demand for e-cigarettes, is that communities, schools and media in China should strengthen health promotion education to prevent e-cigarette use and inform the public, both users and non-users, about the health risks associated with e-cigarette use. The government should launch educational campaigns that target the teenagers and non-smokers who tend to use e-cigarettes out of curiosity. Information should also be disseminated to the cigarette smokers who intend to quit smoking because studies show that this group is susceptible to receiving the wrong information about e-cigarette use, i.e. e-cigarette use helps smoking cessation. On the other hand, to regulate the supply side, the Chinese government should strengthen the auditing of e-cigarette advertisements, avoid the exaggeration of the effectiveness and health ‘benefits’ of electronic cigarettes through advertisements, and avoid the promotion of e-cigarettes to the under-aged population and non-cigarette smokers. Referring to the policies of other countries, the Chinese government should formulate the regulations of e-cigarettes and publish the standards for e-cigarette production as soon as possible.

To summarize, this systematic review indicates that e-cigarette awareness, prevalence, and use prevalence have increased and the use of e-cigarettes may pose harm to the public health. Up until 2018, some provinces and cities in China had launched measures of ‘e-cigarette smoking ban’ independently. For example, Hang Zhou government prohibits e-cigarette use in the smoke-free environment. It is still a long way to go for China’s government to enact effective national policies to address the increasing use of e-cigarettes amid sparse credible evidence from research.

## CONCLUSIONS

We investigated the awareness of e-cigarettes in China and the existing regulation for e-cigarette use and market. Twenty-one studies were identified, and we provided a comprehensive analysis of the e-cigarette awareness ratio in different regions of China and compared the awareness ratios for gender, age group, and smoking status. We also traced e-cigarette use and risk factors, and the relationship between e-cigarette use and smoking cessation. The prevalence of e-cigarette use in China was found to be increasing. The lack of regulation on e-cigarette use and unrestricted e-cigarette marketing practices have fueled consumption of e-cigarettes and misconceptions about the ‘benefits’ of e-cigarettes, such as being aids to help cigarette smokers to quit or reduce smoking, less harmful than traditional cigarettes, and an enhancement of personal image. Given the rising prevalence of e-cigarette use and the detrimental effects of misleading information by e-cigarette companies, marketers, and advertisers, it is crucial that the government of China prioritize the establishment and implementation of regulations for e-cigarette use and market.

## CONFLICTS OF INTEREST

Authors have completed and submitted the ICMJE Form for Disclosure of Potential Conflicts of Interest and none was reported.

## References

[1] Wang F S, Chen L Y, Zhang M M (2017). Electronic Cigarette - From Made in China to Created in China. Consumer Electronics.

[2] Levy DT, Yuan Z, Li Y (2017). The Prevalence and Characteristics of E-Cigarette Users in the U.S. Int. J. Environ. Res. Public Health.

[3] Farsalinos KE, Poulas K, Voudris V, Le Houezec J (2016). Electronic Cigarette Use in the European Union: Analysis of a Representative Sample of 27460 Europeans from 28 Countries. Addiction.

[4] Feng G, Nan Y, Jiang Y (2018). Prevalence of e-cigarette in China: Preliminary findings from two surveys. Tob Induc Dis.

[5] Liang XF (2016). Survey Report on Adult Tobacco in China.

[6] Xia YH, Hu XY, Zhang JY Effects of electronic cigarette on human health and progress in its control. Public Health in China.

[7] Demick B A high-tech approach to getting a nicotine fix [EB/OL].

[8] Shan J (2015). E-cigarette controls considered for safety.

[9] (2015). [Analysis of e-cigarettes market status in China and trend forecasting, 2015-2020].

[10] Barboza D China’s e-cigarette boom lacks oversight for safety [EB/OL].

[11] Tobaco Control Resource Center (2018). International Tobacco Control Policy Assessment Project ITC China Project Report - Findings from Wave 1 to Wave 5 (2006-2015).

[12] O’Neill M How China is lighting up the e-cigarette market [EB/OL].

[13] Miao Zhang Hangzhou city upgrades anti-smoking legislation: Electronic cigarettes are prohibited in public.

[14] State Administration of Market Supervision and Administration and the State Tobacco Monopoly Bureau Circular of the State Administration of Market Supervision and Administration and the State Tobacco Monopoly Bureau on the Prohibition of the Sale of Electronic Cigarettes to Minors.

[15] Moola S, Munn Z, Tufanaru C, Aromataris E, Munn Z (2017). Systematic reviews of etiology and risk. Joanna Briggs Institute Reviewer’s Manual.

[16] Jiang N, Chen J, Wang MP (2016). Electronic cigarette awareness and use among adults in Hong Kong. Addict Behav.

[17] Li W, Jiang GH, Xu ZL (2016). Survey Results Analysis of the use of electronic cigarette among Tianjin resident. Chinese Journal of Prevention and Control of Chronic Diseases.

[18] Wang XS, Zhang XL, Xu XX (2018). Electronic cigarette use and smoking cessation behavior among adolescents in China. Addict Behav.

[19] Li SS, Xiao D, Zhu SL (2015). Survey on the use of electronic cigarettes among smokers in Beijing. Chinese Journal of Clinicians.

[20] Wang MP, Li WHC, Jiang N (2015). E-Cigarette Awareness, Perceptions and Use among Community-Recruited Smokers in Hong Kong. PLoS ONE.

[21] Yao M, Liang SL, Li YZ (2015). Present situation and multivariate analysis of electronic smoking among junior high school students in Guangxi province in 2013. Journal of Applied Preventive Medicine.

[22] Xiao L, Parascandola M, Wang C (2018). Perception and Current Use of E-cigarettes Among Youth in China. Nicotine Tob Res.

[23] Leung L, Ho S, Chen J, Wang M, Lam T (2018). Favourable Perceptions of Electronic Cigarettes Relative to Cigarettes and the Associations with Susceptibility to Electronic Cigarette Use in Hong Kong Chinese Adolescents. International Journal of Environmental Research and Public Health.

[24] Wang MP, Ho SY, Leung LT, Lam TH (2015). Electronic cigarette use and its association with smoking in Hong Kong Chinese adolescents. Addict Behav.

[25] Chen PC, Chang LC, Hsu C, Lee YC (2019). Electronic Cigarette Use and Attempts to Quit Smoking Cigarettes Among Adolescents in Taiwan. J Adolesc Health.

[26] Zhao L, Wang J (2018). E-cigarettes use among urban male tobacco smokers age 15 years or older in China. Tob Induc Dis.

[27] Yao T, Jiang N, Grana R, Ling P, Glantz SA (2014). A content analysis of electronic cigarette manufacturer websites in China. Tob Control.

[28] Jiang N, Ho SY, Lam HL (2017). Electronic cigarette marketing tactics in mainland China. Tob Control.

[29] Li L, Zhou NB, Qu XH (2018). Market Development and Legal Supervision of New Tobacco Products. China Tobacco Journal.

[30] Socrates W, Man W, William L, Kwong A, Lai V, Lam T (2018). Does Electronic Cigarette Use Predict Abstinence from Conventional Cigarettes among Smokers in Hong Kong?. Int J Environ Res Public Health.

[31] Eric FN (2016). On China’s Electronic Tobacco Supervision. Excellent Papers Compilation of China Tobacco Society 2016 - Theme of Tobacco Laws and Regulations. China Tobacco Society.

[32] National Public Service Platform for Standards Information National standards for electronic cigarette.

[33] Fan MJ, Zhao L, Cui HP (2018). Advances in the study of chemical risk in electronic cigarettes. China Tobacco Journal.

[34] (2014). [The harmless theory of electronic cigarettes is unreliable].

[35] Cheung YTD, Wang MP, Ho SY (2017). Public Support for Electronic Cigarette Regulation in Hong Kong: A Population-Based Cross-Sectional Study. Int J Environ Res Public Health.

[36] Xu Y, Guo Y, Liu K, Liu Z, Wang X (2016). E-Cigarette Awareness, Use, and Harm Perception among Adults: A Meta-Analysis of Observational Studies. PLoS ONE.

[37] (2017). TNS opinion & social, Wave EB87.1, Special Eurobarometer 458. Attitudes of Europeans towards tobacco and electronic cigarettes.

[38] Pericot-Valverde I, Gaalema DE, Priest JS, Higgins ST (2017). E-cigarette awareness, perceived harmfulness, and ever use among U.S. adults. Prev Med.

[39] Gallus S, Lugo A, Pacifici R, Pichini S, Colombo P, Garattini S, La Vecchia C (2014). E-cigarette awareness, use, and harm perceptions in Italy: a national representative survey. Nicotine Tob Res.

[40] Centers for Disease Control and Prevention (2016). Tobacco Use Among Middle and High School Students--United States, 2011-2015. MMWR Morb Mortal Wkly Rep.

[41] Kamat AD, Van Dyke AL (2017). Use of Electronic Nicotine Delivery Systems Among Adolescents: Status of the Evidence and Public Health Recommendations. Pediatric Annals.

[42] Kilibarda B, Krstev S, Milovanovic M, Foley K (2018). E-cigarette use in Serbia: Prevalence, reasons for trying and perceptions. Addictive Behaviors.

[43] Hong H, McConnell R, Liu F, Urman R, Barrington-Trimis JL (2018). The impact of local regulation on reasons for electronic cigarette use among Southern California young adults. Addictive Behaviors.

[44] Kim AE, Lee YO, Shafer P, Nonnemaker J, Makarenko O (2013). Adult smokers’ receptivity to a television advert for electronic nicotine delivery systems. Tobacco Control.

[45] WHO Framework Convention on Tobacco Control Electronic nicotine delivery systems.

[46] Food and Drug Administration, HHS (2016). Deeming Tobacco Products To Be Subject to the Federal Food, Drug, and Cosmetic Act, as Amended by the Family Smoking Prevention and Tobacco Control Act; Restrictions on the Sale and Distribution of Tobacco Products and Required Warning Statements for Tobacco Products. Final rule. Federal Register.

[47] U.S Food & Drug Administration Statement from FDA Commissioner Scott Gottlieb, M.D., on Administration’s request for new FDA funding to promote innovation and broaden patient access through competition.

[48] European Commission Questions & Answers: New rules for tobacco products.

