# Erratum: Electronic cigarette use in China: Awareness, prevalence and regulation

**DOI:** 10.18332/tid/118922

**Published:** 2020-03-12

**Authors:** European Publishing Production Team

**Affiliations:** 1 European Publishing, Heraklion, Greece

**Keywords:** electronic cigarettes, awareness, prevalence, regulation, China

**By Wenyuanyue Wang, ZiAn He, Nannan Feng, Yuyang Cai Tobacco Induced Diseases, Volume 17, Issue April, Pages 1-11 Publish date: 16 April 2019**

**DOI:** https://doi.org/10.18332/tid/105393

**In the original article, the acronym ITC was mistakenly defined as the International Trade Center. The correct definition for the acronym ITC is International Tobacco Control. The correction is shown below (Introduction, 2nd page, 3rd paragraph):**

However, the International Tobacco Control (ITC) China survey revealed that the percentage of smokers who had heard of e-cigarette rose from 29% (wave 3; 2009) to 60% (wave 5; 2014).

