# Supplementary material for: Electronic cigarette use in China: Awareness, prevalence and regulation
Source: Tob Induc Dis. 2019 Apr 16;17:30. doi: 10.18332/tid/105393 (PMC6662798; doi:10.18332/tid/105393)
Supplement: Supplementary file 1 [file TID-17-30-s1.pdf]

Appendix 1 Literature included I

Study	Investigation Method	Area Setting	Sample	Measure Methods	Reported Results	Quality
Jiang et al. (2016)[16]	Computer -assisted telephone interviews	Hong Kong China	2419 respondents. Chinese adults aged 15–65 years in Hong Kong since 2013	Chi-square tests to compare e-cigarette awareness and ever use by demographics and cigarette smoking status. Multivariable logistic regression to examine if e-cigarette awareness was associated with gender, age, education and cigarette smoking status with mutual adjustment.	About 75.4% of the respondents had heard of e-cigarettes. Males were more aware than females (80.4% vs. 71.2%, p = 0.04). E-cigarette awareness marginally increased with education level (p = 0.06). The awareness was similar across age (p = .20) and cigarette smoking subgroups (p = 0.51). The multivariable logistic regression model confirmed that greater awareness was associated with male gender.	High quality
Li et al. (2016)[17] *	Questionnaire	Tianjin China	2500 residents aged 15 and over who are non-collective residents who live in the urban area of Tianjin are surveyed, ignoring their hukou and nationality.	Chi-square test for enumeration data	862 (43.6%) people have heard of e-cigarettes, 45 people (2.3%) have used e-cigarettes in the past, and the number of people using e-cigarettes now is 10 (0.5%), all of whom are smokers.	High quality
Wang et al. (2018)[18]	Questionnaire in a mobile-app	China	2042, or 11.56% were between the age of 12 and 18	A logistic regression analysis was conducted to analyse data.	Nearly 90% of the respondents heard of e-cigarettes.Over a quarter of the respondents (26.44%) had ever used e-cigarettes, although only 6.37% were frequent users (20 times and more),9.01% used < 20 times and 11.07% used once or twice.	High quality
Li et al. (2015)[19] *	Questionnaire	Beijing China	956, age > 20 years old, regular smoking with more than 100cigarettes (daily smoking and fixed smoke) and occasional smokers	Measurement data are expressed as mean ± standard deviation, count data is expressed as number of cases or percentage (%), and comparison of rates is performed using χ^2test.	122 (1. 8%) of the smokers used e-cigarettes. 545 smokers (57.0%) have heard of them but they have not used e-cigarettes, and 289 smokers (30.2%) have never heard of e-cigarettes. Using the chi-squared test, there was no significant	High quality

					difference in the awareness rate of e-cigarettes among rural and urban smokers.	
Wang et.al[20]	Questionnaires and follow-up telephone interview	Hong Kong China	1307smokers	STATA 13.0was used for dataanalysis. Multivariate logistic regression was used toyield oddsratios(ORs) for ecigarette awareness,use and perceived effectiveness.	Mostsmokers(82.6%) had heard about e-cigarettes,and13.3% ever used e-cigarettes.Most users (74.1%)and non-users (91.2%) did not perceive e-cigarettes as effective in quitting. Being younger and having a larger family income were associated with e-cigarette awareness. Being younger, a tertiary education and a stronger addiction to nicotine were associated with e-cigarette use,which was itself associated with lower levels of intention to quit and had no association with attempts to quit (p for trend 0.45). E-cigarette use, the last quit attempt being a month earlier,having made a quit attempt lasting 24 hours or longer and perceiving quitting as important were all associated with the perceived effectiveness of e-cigarettes in quitting (all $p<0.05$).	High quality
Yao,et al. (2015)[21] *	Questionnaire	Guangxi China	5,892 junior high school students	The χ^2test was used to compare the count data, and the multivariate unconditional logistic regression analysis was used for multivariate analysis. P <0.05 was considered statistically significant ($p<0.05$).	The proportion of junior high school students who have heard of e-cigarettes is 46.71% (2 736/5 858). The smoking rate of e-cigarettes among junior high school students was 2.85% (167/5 858), with more boys than girls.	Medium quality
Xiao et al.[22]	The global standard questionnaire	Mainland China	A total of 155 117 students from 1020 schools in 31 provinces completed the questionnaire, including 80 357 boys and 74 760	A chi-squared test was used for comparing the differences between groups. Logistic regression was used to explore the factors associated with e-cigarette use and the relationship	About 45.0% of middle school students had heard of e-cigarettes, but only 1.2% reported using e-cigarettes in the last 30 days. Among never-smokers, e-cigarette users were more likely to intend to use a tobacco product in the next 12 months than	High quality

			girls.	between e-cigarette and tobacco use. A p value less than 0.05 was considered statistically signifcant	nonusers (adjusted odds ratio [OR] = 6.970, 95% confidence interval [CI] = 4.474% to 10.857%), and more likely to say that they would enjoy smoking a cigarette (adjusted OR = 14.633, 95% CI = 11.328% to 18.902%)	
Leung et al. (2018)[23]	Anonymous questionnaire	Hong Kong China	Secondary 1 to 6 (U.S. grade 7–12) students (95% response rate) of 92 schools randomly selected from all the 18 districts in Hong Kong.	Descriptive data were weighted by the Education Bureau. Odds ratios yielded from logistic regression may overestimate the associations; prevalence ratios (PRs, ratio of 2 prevalences)were calculated using Cox regression.	The prevalence of ever cigarette smoking was 12.7% (95% CI 12.3–13.0%), including 1.7% (95% CI 1.6–1.9%) ex-smokers,and ever E-cig use was 8.9% (95% CI 8.6–9.2%).<br>Many students (47.2%) had at least 1 favourable perception of E-cig relative to cigarettes, including 24.1% having 1–2, 13.6% having 3–4, and 9.5% having 5 or more favourable perceptions, while less than one-third (28.9%) did not know E-cig.	High quality
Wang et al. (2015)[24]	Questionnaire	Hong Kong China	45,128 students from 75 randomly selected schools (school response rate 20%)	Adjusted odds ratios (AORs) for intention to smoke by e-cigarette use in students with various smoking status were calculated using logistic regression adjusting for potential confounders and clustering effect of schools (to correct intra-class correlation errors).	The prevalence of e-cigarette use (past 30 days) was low overall(1.1%, 95% CI 1.0%–1.2%), but increased sharply with cigarette smoking status: never smokers 0.13%,experimenters 2.02%, former smokers 9.60% and current smokers 9.62%.<br>E-cigarette use was associated with intention to smoke with an AOR (95% CI) of 1.74 (1.30–2.31) adjusting for potential confounders and smoking status.	High quality
Chen et al. (2018)[25]	Questionnaire	Taiwan China	Adolescents aged 12 to 18 years,from the Taiwan Global Youth Tobacco Survey in three years.The weighted sample of adolescents that had used cigarettes in the previous 30 days	The chi-squared test was used to compare the proportions of demographic characteristics among individuals who attempted to quit and those who did not attempt to quit. Logistic regression was used to identify the factors influencing quit	the prevalence of current e-cigarette increased substantially from 9.82%, in 2014 to 28.68% in 2015, followed by a marginal decrease to 27.46% in 2016.<br>The percentages of these smokers who had attempted to quit smoking cigarettes in the previous 12 months were as follows: 71.31% in 2014, 67.69% in 2015,and	High quality

			was as follows: 109,074 (2014), 104,095 (2015), and 90,829 (2016)	attempts. Statistically significant results were reported at $p < 0.05$ or less.	70.59% in 2016.<br>Quit attempts were significant less likely to occur in 2015 and 2016 than in 2014.	
Zhao [26]	data from China City Adult Tobacco Surveys	Mainland China	14 major Chinese cities individually in 2013-14 with 15,008 males aged ⩾15 years	We calculated the prevalence of e-cigarette use among male current tobacco smokers. Multivariate logistic regression was used to assess determinants of e-cigarette use, including age, education, quit attempts in the past 12 months, cigarettes smoked per day, and monthly expenditure on cigarettes.	Among urban male current smokers in China, 3.6% were current e-cigarette users. The likelihood of being current e-cigarette users was significantly higher among: smokers aged 15-29 (OR=2.5) or 30-49 (OR=2.1) years than 50+ years; those who attempted to quit than those who made no attempt (OR=4.5); those with ⩾college education than those with ⩽high school education (OR=2.6); and those who smoked ⩾15 cigarettes per day (OR=2.7) than those who smoked 1-14 cigarettes per day (all p< 0.05).	High quality
Wu et al. (2018)[30]	Questionnaire	Hong Kong China	Smokers who were residents of Hong Kong, aged 18 or above from the 5th ‘Quit to Win’ contest.	Multiple logistic and linear regressions adjusted odds ratios (AOR) and raw coefficient b for quitting and smoking behaviors at the 6-month follow-up in relation to ever EC use at 1 week using Stata13.3.	Ever E-cig users (n = 163) had a higher median number of cigarettes per day, and more of them had a high level of nicotine dependence, while fewer had no previous cessation attempt, compared with non-users<br>(all ps < 0.05). They also rated successful abstinence from conventional cigarettes as more important(p = 0.01) but held similar perceptions on confidence and difficulties of successful quitting.	High quality
Cheung et.al[35]	two-stage, randomized cross-sectional telephone-based survey	Hong Kong China	Adults (aged 15 years or above) who were never smoking (n = 1706), ex-smoking (n = 1712) or currently smoking (n = 1834) were included	Cohen’s effect size (ω) was used to compare the distribution of sex, age, marital status and smoking status of our weighted sample to the general population. The overall distributions of awareness, use, perceived harmfulness of e-cigarettes, and	Over half (57.8%) supported all the four regulations. Banning of e-cigarette promotion and advertisement (71.7%) received slightly less support than the other three regulations (banning of e-cigarette use in smoke-free venues (81.5%); banning of e-cigarette sale to minors (93.9%); sale restriction of nicotine-free e-cigarettes	High quality

	support for the four e-cigarette regulations were analyzed by weighted descriptive statistics. Due to the high prevalence in supporting the regulations, we used four separate Poisson regression models with robust error variance to estimate prevalence ratios for the association of the explanatory variables and the public's support for the four regulations, controlling for socio-demographic variables. All data analyses were conducted using STATA	(80.9%)).

* The original text of the document was in Chinese.

© 2019 Wang W.
