# Supplementary material for: Electronic cigarette use in China: Awareness, prevalence and regulation
Source: Tob Induc Dis. 2019 Apr 16;17:30. doi: 10.18332/tid/105393 (PMC6662798; doi:10.18332/tid/105393)
Supplement: Supplementary file 2 [file TID-17-30-s2.pdf]

Appendix 2 Literature included II

| Study | Focused Area | Reported Conclusion | Quality |
|---|---|---|---|
| Liang et al (2016)[5] * | China | The prevalence rate of e-cigarettes in China is lower than that in Europe and America. In 2015, the population utilization rate was 0.5%, and the adult utilization rate was 3.1%. Most of them were used occasionally. | High quality |
| Xia et al. (2018)[6]* | China | China is the largest production base for e-cigarettes. E-commerce is very developed. Due to the lack of supervision, netizens are exposed to an environment of unrestricted, convenient purchase, low price, and tempting propaganda. | Middle quality |
| Yao et al. (2016)[27] | China | Analysis of 14 categories of slogans from 12 e-cigarette manufacturers in 18 websites in China, among which 89% claimed to be good for health, 78% declared no second-hand smoke exposure, 67% claimed to be able to quit smoking, and used celebrity propaganda to create a variety of styles and styles. | High quality |
| Jiang Nan[28] | Mainland,China | China produces 41% and consumes >38% of the world's cigarette. According to the most recent data of the International Tobacco Control Survey, only 2% of adult current or former smokers in China had ever used e-cigarettes in 2009. China has no regulations on e-cigarettes, including on their manufacture, distribution, sales, health warnings, packaging and advertising. | Middle quality |
| Li et al. (2018)[29]* | China | China Tobacco's industrial enterprises have taken the initiative to increase investment in the development of new tobacco products, actively do a good job in technology and product reserves, and participate in international market competition, and have achieved certain results. China should clarify the positioning of new tobacco products and improve relevant regulatory laws and regulations. | High quality |
| Eric A. Feldman[31]* | China | Regulatory theory and the Chinese legal and institutional environment suggest two approaches，to regulate e-cigarettes as pharmaceuticals or tobacco products，both of which inevitably involve policy weighing and trade-offs. | High quality |
| Fan et al. (2017)[33]* | China | The chemical risks in the smoke liquid mainly include inaccurate identification of nicotine content, aldehydes and ketones, volatile compounds, tobacco-specific nitrosamines and metal elements.<br>The main risks involved in aerosol research include nicotine release and stability, aldehydes, volatile compounds, metallic elements and tobacco-specific nitrosamines.<br>Hazardous substances in e-cigarette smoking materials may migrate into tobacco liquid, aerosols and oral cavity, increasing the health risks of consumers. | High quality |

| | | | |
|---|---|---|---|
| Unknown[34]* | China | In China, e-cigarettes are neither medicines nor health products, medical devices, nor tobacco. Therefore, most e-cigarettes are in the ‘three nos’ state, that is, no product standards, no quality supervision, no safety evaluation. | Low quality |

*The original text of the document was in Chinese

© 2019 Wang W.
